# Study on the Influence of Coil Arrangement on the Output Characteristics of Electromagnetic Galloping Energy Harvester

**DOI:** 10.3390/mi14122158

**Published:** 2023-11-26

**Authors:** Lei Xiong, Shiqiao Gao, Lei Jin, Yaoqiang Sun, Xueda Du, Feng Liu

**Affiliations:** 1 State Key Laboratory of Explosion Science and Technology, Beijing Institute of Technology, Beijing 100081, China; 13781786703@163.com (L.X.); 18712761710@163.com (Y.S.); 3120195090@bit.edu.cn (X.D.); 2 School of Intelligent Manufacturing, Nanyang Institute of Technology, Nanyang 473004, China; 15838776438@163.com

**Keywords:** coils arrangement, electromagnetic, output characteristics, galloping, energy harvester

## Abstract

The arrangement of the induction coil influences the electromagnetic damping force and output characteristics of electromagnetic energy harvesters. Based on the aforementioned information, this paper presents a proposal for a multiple off-center coil electromagnetic galloping energy harvester (MEGEH). This study establishes both a theoretical model and a physical model to research the influence of the position and quantity of the induction coils on the output characteristics of an energy harvester. Additionally, it conducts wind tunnel tests and analyzes the obtained results. With the increase in the number of induction coils, there is a significant improvement in the duty cycle and output power of the MEGEH, resulting in an amplified energy conversion efficiency. At a wind speed of 9 m/s, the duty ratios of a single set of coils (SC), two sets of coils (TC), and multiple sets of coils (MC) are 30%, 51%, and 100%, respectively. The total output powers are 0.4 mW, 0.62 mW, and 0.72 mW. However, the rate of output growth has decreased from 55% to 16%. The position of the coils affects the initial electromagnetic damping of the energy harvester. Changing the position can reduce the initial electromagnetic damping, thereby decreasing the critical wind speed. The critical wind speed of the MEGEH decreases as the induction coil is positioned further away from the vibration center. When the distance is sufficiently large, the electromagnetic damping force becomes negligible. When the induction coil is positioned centrally, the MEGEH demonstrates its maximum critical wind speed, which has been measured at 4.01 m/s. When the initial distance between the induction coil and the vibrating component is increased to 10 mm, the critical wind speed reaches its minimum value of 2.23 m/s. Therefore, it is necessary to optimize the arrangement of the coils. The coils of the MEGEH should be arranged with the MC and a 10 mm offset from the center.

## 1. Introduction

Wind-induced vibration energy harvesting is a methodology utilized to capture energy by exploiting the vibrations of a bluff body resulting from the force exerted by wind. The conversion of vibration energy into electrical energy is achieved through a variety of mechanisms. There are various methods for harnessing energy, such as vortex vibration energy harvesting [1,2,3], flutter energy harvesting [4,5], and multi-vibration compound energy harvesting [6,7]. Galloping vibration is a specific form of wind-induced vibration that is distinguished by its large amplitude. It was initially proposed by Den Hartog in the analysis of atypical oscillations in transmission lines and has since been further developed through ongoing research and investigation by G.V. Parkinson [8,9] and others, having made notable progress in the development of the galloping theory over the years. A. Barrero-Gil et al. [10] were the first to propose the utilization of cross-flow galloping as a method for converting and harnessing energy from fluid flows. They developed a theoretical model for the potential of galloping energy harvesting. They also conducted preliminary experimental studies to investigate the influence of cross-sectional geometry on the efficiency of energy harvesting. Furthermore, they successfully demonstrated the feasibility of the galloping energy conversion technique. Subsequently, there has been increasing interest among researchers in the field of vibration energy harvesting technology, leading to the emergence of numerous innovative designs and enhancement strategies.

The galloping energy harvester converts wind energy into electric energy and outputs it through electromagnetic induction [11,12], piezoelectric conversion [13], and piezoelectric electromagnetic cooperation [14]. Currently, studies on galloping energy harvesters can be broadly categorized into two main groups. On one hand, there are studies on the structural aspects of the vibration unit of the energy harvester, including the influence of the cross-sectional shape of the bluff body [15] and the design of the cantilever beam [16] on the electric energy output. Jayant Sirohi and Rohan Mahadik [17,18] proposed a piezoelectric electromechanical coupling model that is based on the quasi-steady state theory. They also devised a prototype for an energy harvesting device, which involved the integration of a triangular cross-section bluff body, with the aim of conducting experimental validation. The experimental results demonstrated a high level of concurrence with the theoretical model. Later, an experimental model with a D-section was developed, and experiments were conducted to obtain the output characteristics of the energy harvester. The results revealed a significant correlation between the velocity of wind and the generated power of the energy harvesting device, with the latter experiencing a rapid increase as the former increased. The result showcases the feasibility of harnessing wind energy through the utilization of a D-section device. Yaowen Yang et al. [19] conducted a comparative analysis of the output characteristics exhibited by square, rectangular, D-shaped, and triangular bluff bodies. When the characteristic dimensions are equal, a bluff body with a square cross-section exhibits superior output characteristics. Therefore, the bluff body used in this paper is also selected as a square section. Lingzhi Wang et al. [16] introduced a novel energy harvester with a cantilever beam of a variable cross-section. An analytical study was conducted to investigate the effect of a single modification in the cross-sectional width, as well as the combined effect of modifying both the cross-sectional width and thickness simultaneously. These alterations led to a noteworthy 61% increase in the power output compared to the energy harvester with a cantilever beam of uniform cross-section. On the other hand, external structures, such as nonlinear magnetic structures [20,21,22] and additional components [23,24], are incorporated to modify the motion of the vibration unit and enhance the output characteristics of the energy harvester. Felix Ewere et al. [25] conducted a study on the output characteristics of a square-section bluff body and a modified energy harvester with shock absorbers. The study revealed that the maximum peak power achieved was 13 mW at wind speeds of 8 m/s. The performance of the output was improved by the addition of three externally fixed magnets, resulting in the creation of a four-stable output through the interplay of forces among them. Hu, J. et al. [23] added a secondary beam to a cantilever beam in order to reduce the oscillation caused by the wind speed and increase the output power of the harvester. This was achieved by adjusting the distance between the secondary beam and the passivated body. Li, C. et al. [26] designed a hollow bluff body, in which a single-degree-of-freedom spring and coil were installed, the magnet was fixed at the free end of the spring, and the vibration of the cantilever beam drove the magnet to vibrate, capturing energy. H.L. Dai et al. [27] established the coupling equation between the lateral vibration of a bluff body and the induced current. The load resistance affects the electromagnetic damping and critical wind speed of the galloping. L.B. Zhang et al. [28] designed an energy harvester with a bluff body of a Y-shaped cross-section. They arranged the external magnets in a Halbach array configuration, which led to an improved output power. H. D. Le and S.-D. Kwon [29,30] first proposed a double-magnet energy harvester and later suggested a structure with magnets arranged in a Halbach array. When the thickness of the magnet is twice that of the coil and the height of the coil is twice that of the magnet, the magnetic induction intensity is maximized. X. Li et al. [31] designed a bistable nonlinear magnetic coupling structure, which consisted of a movable magnet, a fixed coil, and two fixed magnets. When the wind velocity is 11 m/s, the starting wind speed decreases by 28%, and the power output increases by 136%. H.L. Dai et al. [32] affixed the magnets onto the cantilever beam and positioned the coils on the lateral side of the beam. A nonlinear electromechanical distribution parameter model was developed, and an investigation was conducted to analyze the effects of the external resistance of the coils and the position of the magnets on the output characteristics of the energy harvester. T. Wacharasindhu and J. W. Kwon [33] studied parameters such as the coil area and the number of turns. The greater the number of turns, the higher the output of the electromagnetic energy harvester.

It can be found that the research on galloping energy harvesters fails to take into account the influence of the coil arrangement on the energy output of the harvester; whether it is focused on the vibration unit or additional external structures. The arrangement of the coils will greatly influence the electromagnetic damping of the energy harvester, thus influencing the critical wind speed and output power of the energy harvester. Therefore, it is imperative to study the influence of the coil on the output of an energy harvester. In view of this problem, this paper proposes an electromagnetic energy harvester with multiple off-center coils. By adjusting the configuration of the induction coils, the initial electromagnetic damping force acting on the MEGEH can be modified. This adjustment leads to an increase in both the duty ratio and output power of the MEGEH. In addition, it also leads to a decrease in the critical wind speed of the MEGEH. Consequently, these modifications contribute to enhancing the output characteristics of the MEGEH.

## 2. Modeling, Analysis, and Experimental Setup

### 2.1. Modeling

**Figure 1:**
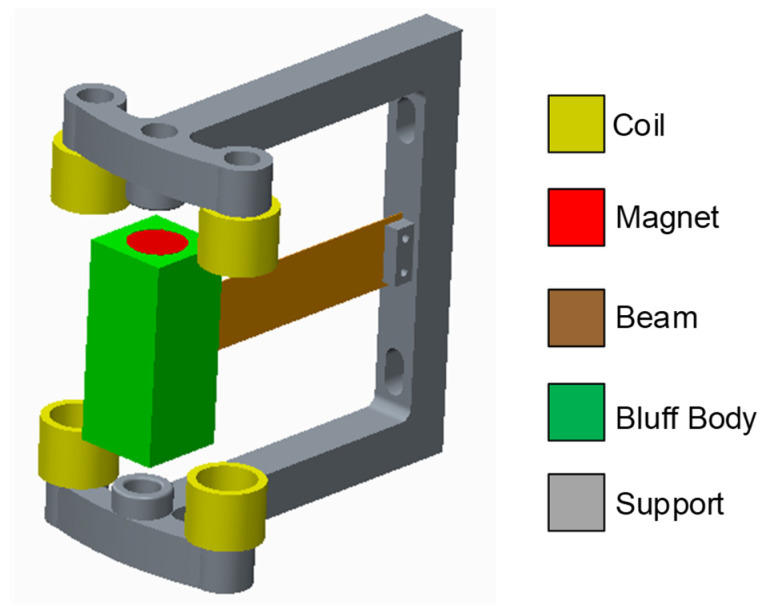
Model diagram of the MEGEH.

The structure of the MEGEH proposed in this paper is shown in Figure 1. It consists of a bluff body with a square cross-section, two cylindrical permanent magnets, an elastic beam, multiple induction coils, and a fixed bracket. One end of the elastic beam is fastened to the bracket using bolts, while the opposite end is affixed to the square bluff body. Two cylindrical permanent magnets are positioned within the central regions of the two ends of the square bluff body. The elastic beam, the bluff body, and the permanent magnet together constitute the vibrating component of the MEGEH. The induction coils are symmetrically affixed to the bracket in both upper and lower positions, and their quantity and placement are adjusted in accordance with the specific testing requirements.

When the wind speed exceeds the critical galloping wind speed of the MEGEH, the vibrating component initiates vibrations, resulting in periodic relative motion between the permanent magnet and the induction coil. The relative motion will generate a changed magnetic field within the coil. As a result, an induced electromotive force and current will be generated within the coil loop. The motion of the vibrating component will be dampened by the induced current. The damping force will change with variations in the relative distance between the permanent magnet and the coil, as well as the velocity of movement. The output characteristics of the vibrating component will be affected as a result. In the present study, the modification of the electromagnetic damping force is achieved by adjusting the quantity and placement of the coils. This optimization aims to augment the output characteristics of the electromagnetic galloping energy harvester, consequently leading to an enhanced overall performance.

### 2.2. Analysis

**Figure 2:**
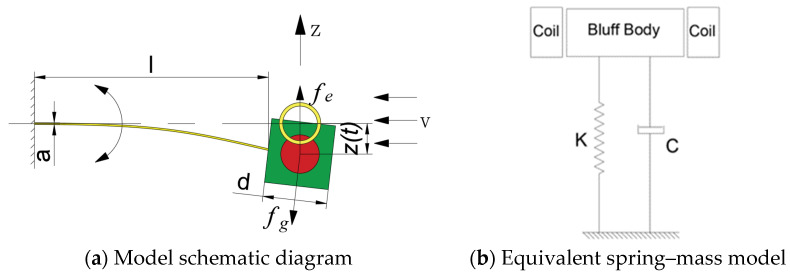
Model schematic diagram and equivalent spring–mass model of the MEGEH.

The model schematic diagram of the MEGEH is depicted in Figure 2a. The vibrating component and induction coil of the galloping model can be regarded as an equivalent spring–mass–damping system when exposed to wind, as shown in Figure 2b. The control equation for the MEGEH can be represented as Formula (1).

(1) $$\{\begin{array}{cc} M\ddot{z}(t)+C\dot{z}(t)+Kz(t)=f_{g}-f_{e} & \left(1-1\right)\\ L_{e}{\dot{i}}_{e}(t)+(R_{e}{+R}_{L})i_{e}(t)+e(t)=0 & \left(1-2\right) \end{array}$$

The variables utilized in the equation are defined as follows: *f_g_* represents the aerodynamic force exerted on the bluff body, *f_e_* represents the electromagnetic damping force generated by the induced current on the bluff body, M represents the equivalent mass of the vibrating component, C and K represent the damping coefficient and elastic coefficient of the elastic beam, R_L_ represents the load resistance, *e*(*t*) represents the induced electromotive force produced by the coils, *i_e_*(*t*) represents the loop current, L_e_ and R_e_ represent the resistance and inductance of the induction coil, respectively.

The output power and critical wind speed are considered as the primary indicators for evaluating the performance of an energy harvester. The lower the critical wind speed, the greater the output power, and the better the performance of the MEGEH. According to Formula (1), when there is no current in the induction coil loop, the electromagnetic damping force *f_e_* is 0, and the vibration unit can gallop only by overcoming the mechanical damping of the structure itself; when there is current in the induction coil loop, the electromagnetic damping force *f_e_* is not zero, and the vibration unit must overcome not only its own mechanical damping but also the electromagnetic damping force before galloping can occur. When the mechanical damping remains constant, the initial damping during vibration is determined by the electromagnetic damping. The greater the electromagnetic damping force, the greater the critical wind speed of the galloping; the smaller the electromagnetic damping force, the lower the critical wind speed of the galloping. This implies that the minimum critical wind speed required for the MEGEH corresponds to the critical wind speed at which galloping occurs when the circuit is open. Therefore, in order to decrease the initial wind speed required for the MEGEH and expand its operational range, it is imperative to minimize the electromagnetic damping force when the MEGEH initiates vibration.

The following is a depiction of the electromagnetic damping force *f_e_*, which is exerted on the MEGEH during galloping.

(2) $$\begin{array}{cc} f_{e}=\frac{\partial W}{\partial z}=\frac{\partial \Phi }{\partial z}i_{e}(t)=\mathrm{NB}\frac{\partial S}{\partial z}i_{e}(t)=g_{e}i_{e}(t) & \end{array}$$

The electromechanical coupling coefficient of the induction coil during energy harvesting is denoted as *g_e_*. N represents the induction coil turns, and B represents the average magnetic induction intensity.

When the MEGEH initiates vibration, the current generated is nearly zero, and the electromagnetic damping force *f_e_* is determined by the electromechanical coupling coefficient *g_e_*. The smaller the electromechanical coupling coefficient *g_e_* is, the smaller the electromagnetic damping force *f_e_* is, which is advantageous for the initiation of galloping. When the radii r of the magnet and the coil are equal, the electromechanical coupling coefficient *g_e_* is given by [34].

(3) $$g_{e}=\{\begin{array}{ll} -\mathrm{NB}\sqrt{4r^{2}-z^{2}(t)} & \left|z(t)\right|\leq 2r\\ 0 & \left|z(t)\right|>2r \end{array}$$

where *z*(*t*) represents the center distance between the vibrating component and the induction coil.

When *z*(*t*) equals zero, indicating the overlap between the magnet and the induction coil, the electromechanical coupling coefficient, electromagnetic damping, and critical wind speed reach their maximum values. The induction coils are in the initial equilibrium position of vibration. When *z*(*t*) ≥ 2r, indicating that the magnet and the induction coil do not intersect, the electromechanical coupling coefficient becomes 0. Consequently, the electromagnetic damping force is approximately 0, and the critical wind speed is minimized. At this time, the induction coil is far from the vibrating component. Therefore, by manipulating the relative positioning of the induction coil and the magnet, it is possible to decrease the electromechanical coupling coefficient during the initiation of vibration in the MEGEH. This, in turn, leads to a reduction in the electromagnetic damping force, resulting in a decrease in the critical wind speed at which galloping vibration occurs in the MEGEH. Consequently, the output characteristics of the MEGEH are enhanced.

The aerodynamic force *f_g_* [35] on the energy harvester during galloping can be approximated as follows:

(4) $$f_{g}=\frac{1}{2}{\rho V}^{2}\mathrm{hd}[A_{1}(\dot{z}/V)+A_{3}{\left(\dot{z}/V\right)}^{3}]$$

where h and d represent the height and cross-sectional side length of the bluff body, respectively. A_1_ and A_3_ are empirical coefficients related to aerodynamics. ρ denotes the air density, and V represents the wind speed.

By incorporating Formulas (2) and (3), and Formula (4) into Formula (1-1), the following expression can be derived.

(5) $$\begin{array}{l} \ddot{z}(t)+\frac{1}{M}\left[C+\frac{g_{e}{}^{2}}{R_{e}{+R}_{L}}-\frac{1}{2}{\rho \mathrm{hdVA}}_{1}-\frac{{\rho \mathrm{hdVA}}_{3}}{2}{\left(\frac{\dot{z}(t)}{V}\right)}^{2}\right]\dot{z}(t)\\ +\frac{K}{M}z(t)=0 \end{array}$$

By disregarding the nonlinear factor of the aerodynamic coefficient and assuming $C+\frac{g_{e}{}^{2}}{R_{e}{+R}_{L}}-\frac{1}{2}{\rho \mathrm{hdA}}_{1}V=0$, the critical wind speed *V_cr_* for galloping energy harvesters can be obtained as follows:

(6) $$V_{cr}=\frac{{2C(R}_{e}+R_{L})+{2N}^{2}B^{2}{(4r}^{2}-z^{2}(t))}{{\rho \mathrm{hdA}}_{1}{(R}_{e}+R_{L})}$$

**Figure 3:**
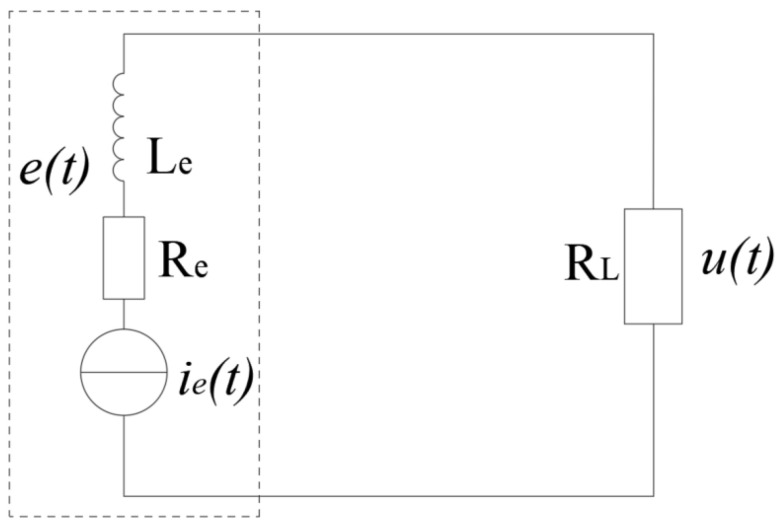
Equivalent circuit diagram of the MEGEH.

The equivalent circuit of the MEGEH is depicted in Figure 3. Since the vibration frequency of the system is low, the influence of inductance can be disregarded. Formulas (1) and (2) can be simplified to Formula (7):

(7) $${(R}_{e}+R_{L})i_{e}(t)+e(t)=0$$

When galloping, the distance between the coil and the magnet in the vertical direction remains unchanged, ignoring the change in the magnetic induction intensity in the horizontal direction. The expression for the induced electromotive force *e*(*t*) in the coil is

(8) $$e(t)=-\mathrm{NB}\frac{\partial S}{\partial t}=-\mathrm{NB}\frac{\partial S}{\partial z}\dot{z}(t)=-g_{e}\dot{z}(t)$$

The load voltage *u*(*t*) is derived by substituting Equation (8) into Equation (7).

(9) $$\begin{array}{cc} u(t)=-\frac{R_{L}}{R_{e}{+R}_{L}}e(t)=\frac{R_{L}}{R_{e}{+R}_{L}}g_{e}\dot{z}(t) & \left|z(t)\right|\leq 2r \end{array}$$

According to Formula (9), the MEGEH will exhibit a voltage output only when the distance between the magnet and the coil is less than 2r. Conversely, if the distance exceeds 2r, the MEGEH will yield an output of 0. Therefore, through the regulation of the maximum distance between the magnet and the coil, it is possible to reduce the duration in which the MEGEH does not generate any output. This enhances both the duty cycle and output power of the harvester.

The comprehensive output power *P* of the MEGEH is

(10) $$P=\sum_{i=1}^{n}p_{i}$$

where *P_i_* represents the output power of each coil.

### 2.3. Experimental Setup and Parameter Identifications

**Figure 4:**
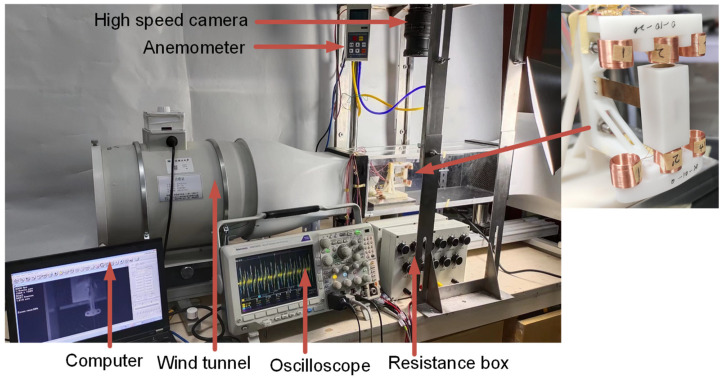
The physical diagram of the testing device.

Figure 4 shows the physical diagram of the testing device. The wind tunnel test equipment includes a KT-03 aerodynamic instrument(produced by Wangai Teaching Equipment Co., Ltd., Shanghai China), a high-speed camera for image capture and an oscilloscope for voltage measurement. Table 1 displays the model’s parameters.

## 3. Experiment and Discussion

The arrangement of the induction coil has a significant impact on the output characteristics of an energy harvester. According to the theoretical analysis results, the duty cycle and initial electromagnetic damping of the MEGEH can be modified by adjusting the quantity and position of the induction coils. Through wind tunnel testing and comparative analysis, the optimal coil arrangement is determined, resulting in an improved energy conversion efficiency for the MEGEH.

### 3.1. Output Response of the MEGEH

The assessment of effectiveness in an energy harvester is primarily determined by a parameter known as output power. Firstly, the objective of this study is to investigate the influence of the coil arrangement on the output voltage and output power of the MEGEH.

#### 3.1.1. The Output Response of the SC

**Figure 5:**
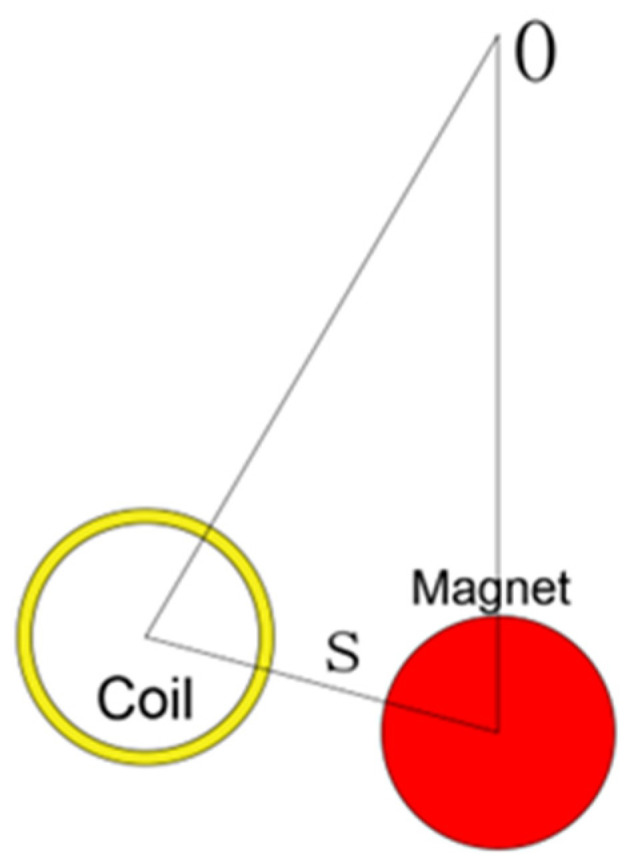
Schematic diagram of the induced coil’s position when it is SC.

**Figure 6:**
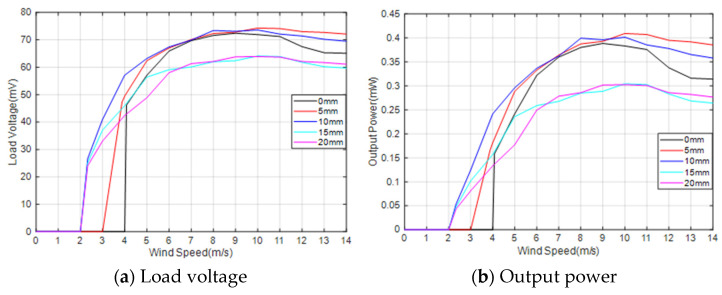
Output comparison of SC at different positions.

In order to investigate the influence of the arrangement of the induction coil on the output characteristics of the MEGEH, the experiment entailed measuring the load voltage of the induction coil at different positions. The two coils, located at both ends of the bluff body, form a pair. The output phases of the two coils in each set are the same, and they are connected in series. The internal resistance of each set is 13.2 Ω. The galloping phenomenon exhibits a symmetrical periodic pattern. Consequently, the SC test is conducted on the left side of the central position, as depicted in Figure 5. The variable S represents the distance between the induction coil and the center of vibration. The load voltages were measured and recorded at different positions of S, specifically S = 0, S = 5 mm, S = 10 mm, S = 15 mm, and S = 20 mm. The voltage and power generated by the MEGEH are depicted in Figure 6. The MEGEH’s output initially increases and then decreases as wind speed increases. When the value of S is less than or equal to 10, the MEGEH exhibits a similar maximum load voltage and output power, measuring 73 mV and 0.4 mW, respectively. However, the critical wind speed and peak voltage occur at different wind velocities. When the value of S exceeds 10, a discernible decline is observed in both the output voltage and power of the MEGEH, resulting in values of 64 mV and 0.3 mW, respectively. When the wind speed is below 9 m/s, the MEGEH attains its maximum load voltage and output power at S = 10. When the wind speed exceeds 9 m/s, the MEGEH experiences the slowest decrease in both the output voltage and output power at S = 5. Therefore, when employing the SC, it is advisable to place the coil off-center, while ensuring that the offset distance is not too large. In this experimental study, a more suitable choice for the offset distance would be 10 mm.

**Figure 7:**
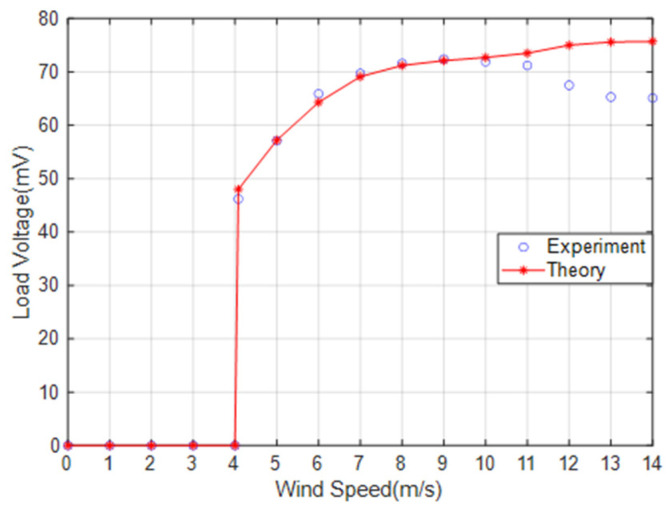
Comparison of the theoretical voltage and experimental voltage of the energy harvester when S = 0.

**Figure 8:**
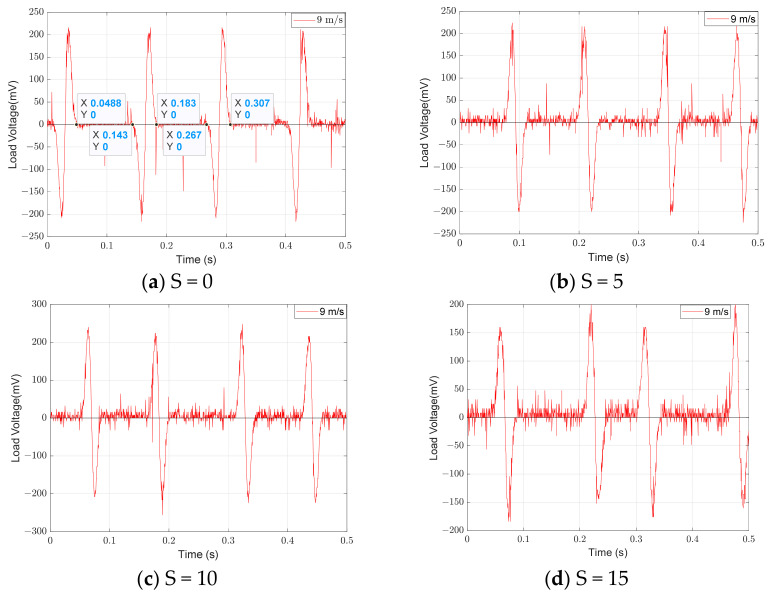
Output waveforms of SC at different positions.

Figure 7 presents a comparison between the theoretical voltage and the experimental voltage output of the energy harvester when S = 0 mm. When the wind speed does not exceed 11 m/s, the error is negligible. However, when the wind speed exceeds 11 m/s, the error gradually increases. The error is 14% when the wind speed is 14 m/s. The main reason for the increased error is the use of “ $\dot{z}/V$” as an approximation for the wind attack angle when calculating the aerodynamic force. The ambient wind speed is generally much lower than 11 m/s, making the theoretical model feasible. The MEGEH with SC exhibits a common characteristic, namely, a small output duty cycle. No matter what the coil offset distance is, it remains consistently around 30%. This means that, during a vibration period, only 30% of the time there is an induced electromotive force, while no conversion of electric energy occurs during the remaining time. Consequently, the energy generated by the vibrations is entirely wasted. When the coils are off-center, the duration of the continuous no-output state lasts longer in each cycle. As depicted in Figure 8, the output voltage of the MEGEH exhibits discontinuity, with prolonged periods of no output observed within each cycle. According to the analysis of Formula (9), it has been determined that the generation of induced electromotive force only occurs when the distance between the induction coil and the magnet is less than 2r. However, the amplitude of the galloping energy harvester is large, which makes the maximum distance between them in excess of 2r, as shown in Table 2. The greater the distance exceeds, the longer the time without any output, and the lower the duty cycle of the MEGEH. Therefore, by reducing the maximum distance between them during the vibration process, it is possible to improve the duty cycle of the MEGEH, thereby leading to an improvement in the output efficiency of the MEGEH.

#### 3.1.2. The Output Response of the TC

**Figure 9:**
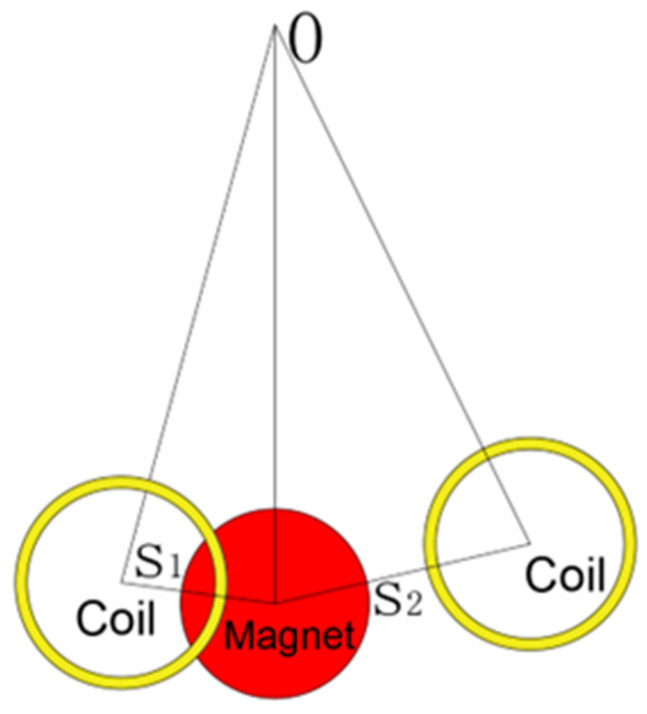
Schematic diagram of the induced coil’s position when it is TC.

In order to improve the duty cycle of the MEGEH and reduce the no-output time, it is imperative to decrease the maximum distance between the coils and the magnets. Therefore, a set of induction coils is added on the opposite side of the central position. The output structure consists of two sets of coils, as illustrated in Figure 9. When the two sets of coils are positioned on either side of the central position, they can be categorized into symmetrical and asymmetrical arrangements. Two layout modes are selected for each type, resulting in a total of four distribution modes being implemented. The distribution of coils is presented in Table 3. The coils utilized in the experiment have an outer diameter of 12.3 mm. In order to mitigate potential interference, the distance between the two sets of coils should be set at S_1_ + S_2_ = 14 mm.

**Figure 10:**
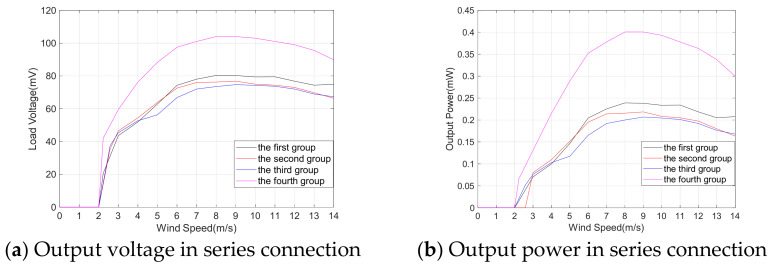

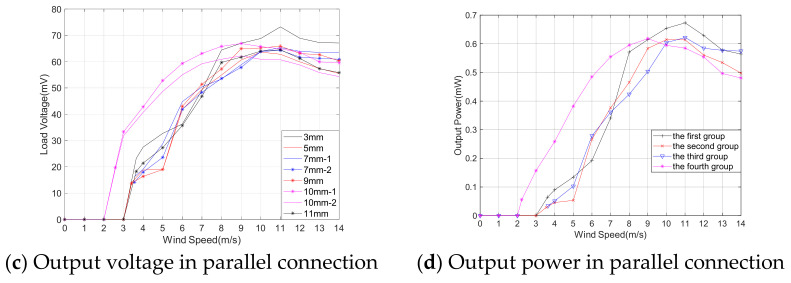
Comparison of output voltage and power when the induced coil is TC.

The selection of the output mode for the MEGEH. Figure 10 depicts the voltage and power characteristics when the TCs are connected in series and in parallel, respectively. When the external resistance of the coil is equal to the internal resistance, the energy harvester achieves its maximum output power. In series, the load resistance is 26.4 Ω. In parallel, the load resistance is 13.2 Ω. Among the figures presented, Figure 10a,b depict the output voltage and power of the TC connected in series. The fourth type consistently demonstrates the highest output voltage and power. Figure 10c,d depict the voltage and total power, respectively, when the TCs are connected in parallel. Although the voltage output of the coils connected in series is higher compared to those connected in parallel, the maximum power output of the former is only 0.4 mW, whereas the power output in parallel is 0.7 mW. The main factor influencing this phenomenon is the distance between the two sets of coils, which is less than 14 mm. When the vibrating component is positioned between the two sets of coils, the simultaneous generation of induced electromotive force occurs in both groups. However, there exists a phase difference between the two induced electromotive forces. Therefore, the parallel output mode of the coils has been selected for this experiment in order to achieve a higher output power.

**Figure 11:**
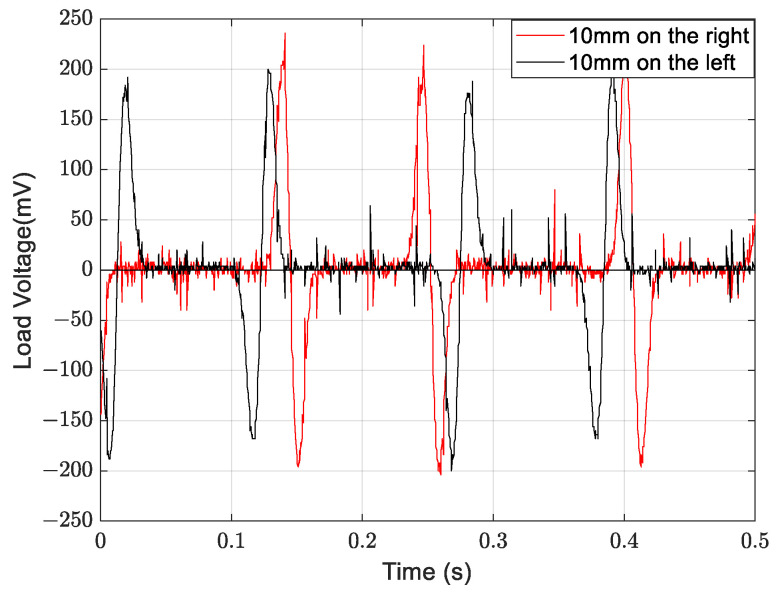
Waveform diagram that illustrates the parallel output of the fourth group of coils.

When the SC is used, the maximum distance between the coil and the magnet is (S + amplitude). When the TC is used, there are additional coils on the opposite side of the equilibrium position. So, the maximum distance between the magnet and the coil is reduced, resulting in a shorter movement stroke beyond 2r. This leads to substantial improvements in both the duty cycle and output power of the MEGEH. Figure 11 presents a waveform diagram that illustrates the parallel output of the fourth group of coils. The output duty ratio is 51%, representing an increase of 70% compared to that of SC. The output power has been enhanced from 0.4 mW to 0.62 mW, representing a 55% increase. The peak voltage of the output is observed to decrease from 210 mV to 185 mV, resulting in a smoother output curve. Additionally, it has been observed that there is a discernible trend of initially increasing and subsequently decreasing the output voltage and power of the induction coil as the wind speed increases. When the wind speed is below 9 m/s, it is evident that the fourth group exhibits superior output voltage and power compared to the other groups. When the wind speed is 5 m/s, the fourth type exhibits an average output voltage of 50.8 mV and a power output of 0.38 mW. The minimum output is the second type, with a voltage of only 19 mV and a power of merely 0.05 mW. This output is 2.67 times lower in voltage and 7.6 times lower in power compared to it. When the wind speed exceeds 9 m/s, the first group exhibits optimal output voltage and power. The average wind speed in the environment is low, only a few meters per second. Therefore, from an engineering application perspective, the energy harvester is most concerned with the output characteristics at low speeds. Therefore, the output characteristics of the fourth group are the best. Compared to the SC, the TC demonstrates a significant improvement in the output power. As shown in Table 4, the output power of the TC demonstrates a significant improvement compared to the SC.

#### 3.1.3. The output Response of MC

**Figure 12:**
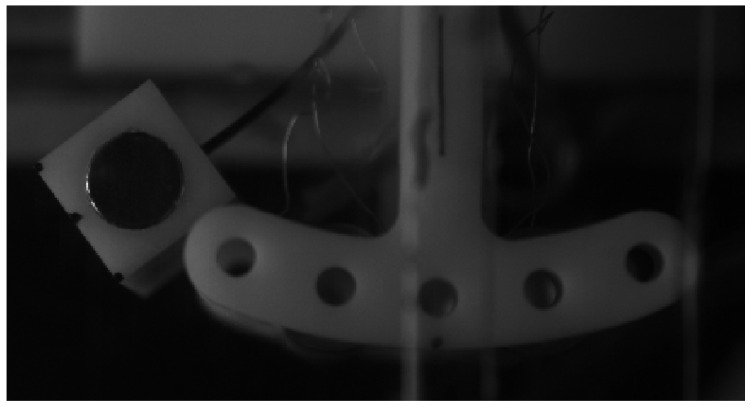
Amplitude of the MEGEH at 11 m/s.

**Figure 13:**
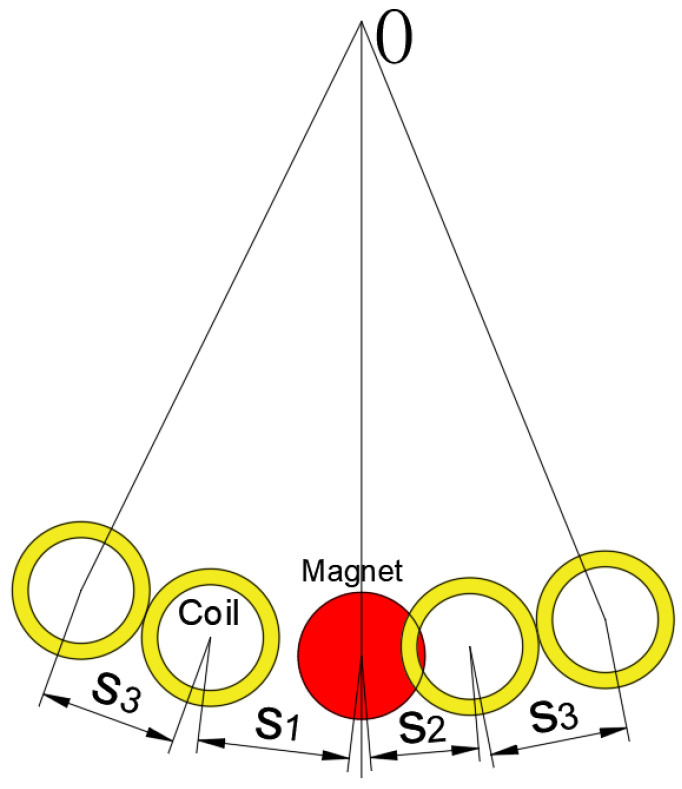
Schematic diagram of the induced coil’s position when it is MC.

Although the utilization of TC greatly improves the duty cycle and output power of the MEGEH, it is noteworthy that the duty cycle remains low during high wind speeds and when the amplitude of the MEGEH is excessively large. The reason for the time without output is determined by the duration of vibration when the amplitude exceeds the coil. As depicted in Figure 12, at a wind speed of 11 m/s, the amplitude of the fourth group has reached 28.6 mm in the Z direction, exceeding the position of the coils entirely. Consequently, an additional set of coils is incorporated outside the existing two sets of coils, and the arrangement of multiple sets of coils is adopted, as illustrated in Figure 13. The arrangement of the middle two sets of coils is the same as that of the aforementioned TC. Additionally, the outer two sets of coils are positioned adjacent to the middle two sets, with a specific distance of S_3_ = 14 mm, as illustrated in Table 5. When the wind speed is at a low level, the amplitude of the MEGEH is small, resulting in output only from the two central sets of coils. Conversely, when the wind speed is high, the amplitude increases and extends to the outer coil and the subsequent generation of induced electromotive force in the outer coil as well. The distance between the two sets of coils on the outer side is far away, resulting in a large output phase difference. Therefore, it is possible to connect these two sets of coils in series and then in parallel with the middle two sets of coils.

**Figure 14:**
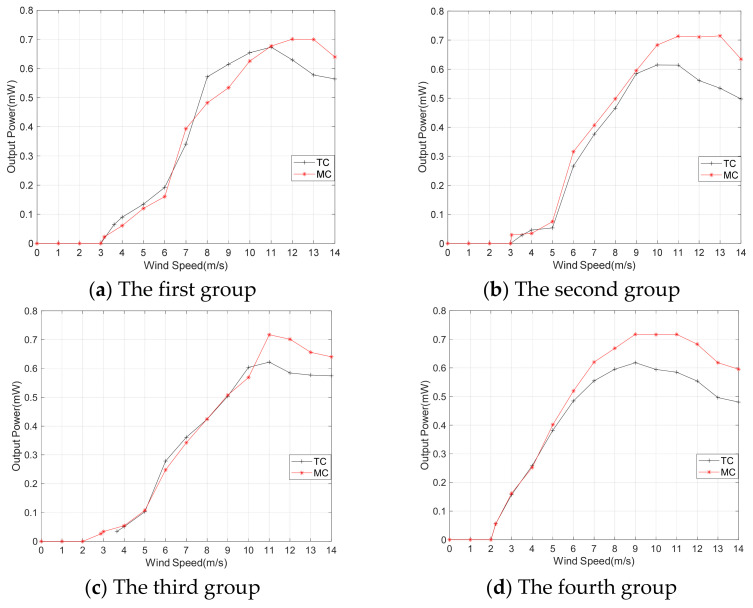
Comparison of the output power between TC and MC.

**Figure 15:**
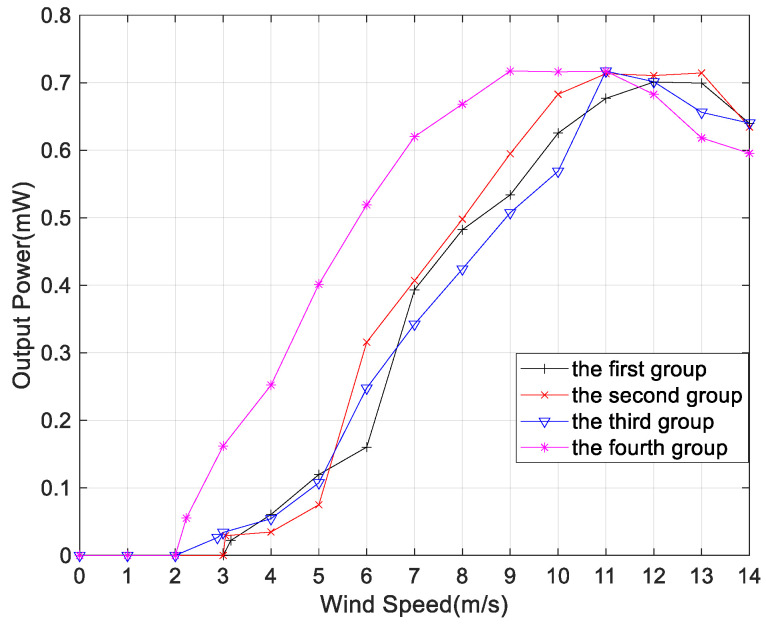
Comparison of the comprehensive output power in four different layout modes.

Figure 14 presents a comparison of the output power between TC and MC, taking into account the different arrangement modes. When the wind speed is at a low level, the amplitude of the MEGEH is small, resulting in the absence of induced electromotive force in the outer coils. The output power of both the TC and the MC is essentially the same. However, when the wind speed reaches 5~6 m/s, the output power of the MC exceeds that of TC. Furthermore, as the wind speed increases, the disparity in the output power gradually becomes larger. The generation of power output by the outer coil is attributed to the increased amplitude of the MEGEH. Additionally, it has been observed that the output of the outer coil exhibits a direct correlation with the wind speed, indicating that higher wind speeds lead to increased output from the outer coil. Furthermore, the utilization of MC in the MEGEH leads to an increased wind velocity at the turning point of the power output curve. According to Formula (9), the output voltage *u*(*t*) is related to the vibration speed. With multiple coils, the energy harvester is subjected to greater electromagnetic damping during a vibration cycle. This results in a higher wind speed when the energy harvester reaches the same vibration speed. This, in turn, leads to a larger amplitude of power and a broader working range. Therefore, the utilization of MC in the MEGEH leads to enhanced efficiency in energy conversion. Figure 15 depicts a comparison of the comprehensive output power among MC in four different arrangement modes. When the wind speed is less than 11 m/s, the fourth output power exhibits the highest value. Therefore, the output power of MC is higher, and the fourth group is more favorable.

### 3.2. Critical Wind Speed of the MEGEH

The critical wind speed is another important parameter in assessing the effectiveness of an energy harvester. According to the aforementioned analysis, the minimum critical wind speed of the MEGEH corresponds to the wind speed at which the coil is in an open state. The measured value for the critical wind speed is 2.23 m/s, suggesting that the MEGEH’s minimum critical wind speed is 2.23 m/s. When the coil is off-center, the critical wind speed *V_cr_* of the MEGEH in Formula (6) can be expressed as follows:

(11) $$V_{cr}=\frac{{2C(R}_{e}+R_{L})+{2N}^{2}B^{2}{[4r}^{2}-{(z(t)-S)}^{2}]}{{\rho \mathrm{hdA}}_{1}{(R}_{e}+R_{L})}$$

**Figure 16:**
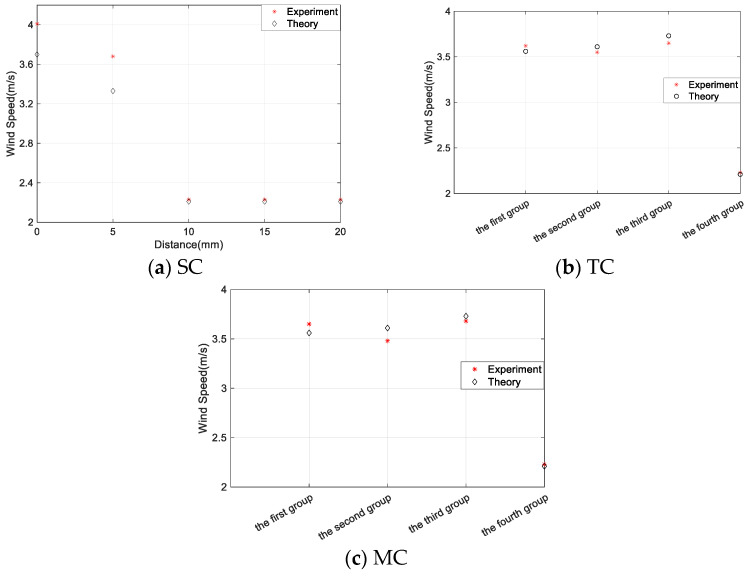
Comparison of the experimental and theoretical critical wind speed of the MEGEH.

Figure 16 presents a comparison between the theoretical and experimental values of the critical wind speed for the MEGEH with the SC (a), TC (b), and MC (c). When utilizing the SC, the MEGEH’s critical wind speed demonstrates a gradual decrease as the distance S increases, ultimately reaching the minimum critical wind speed. When S = 0, the critical wind speed of the MEGEH is the largest, which is 4.01 m/s; when S = 5, the critical wind speed of the MEGEH is 3.68 m/s; when S ≥ 10, the critical wind speed of the MEGEH is 2.23 m/s. The reason for this is that the induced current generated by the MEGEH creates an electromagnetic damping effect on its vibration. The magnitude of the damping force is directly influenced by the distance between the induction coil and the magnet. The electromagnetic damping force increases as the distance decreases and decreases as the distance increases. When the distance is large enough, the effect of the electromagnetic damping force can be approximately zero. In the case of the MC, the electromagnetic damping force can be ignored because the outer coils are far away, so the critical wind speed is basically the same as that in the case of the TC. Additionally, the critical wind speed in the fourth arrangement mode is the smallest, which is 2.23 m/s. Therefore, by altering the placement of the induction coil, it becomes feasible to decrease the MEGEH’s critical wind speed to its minimum value.

**Figure 17:**
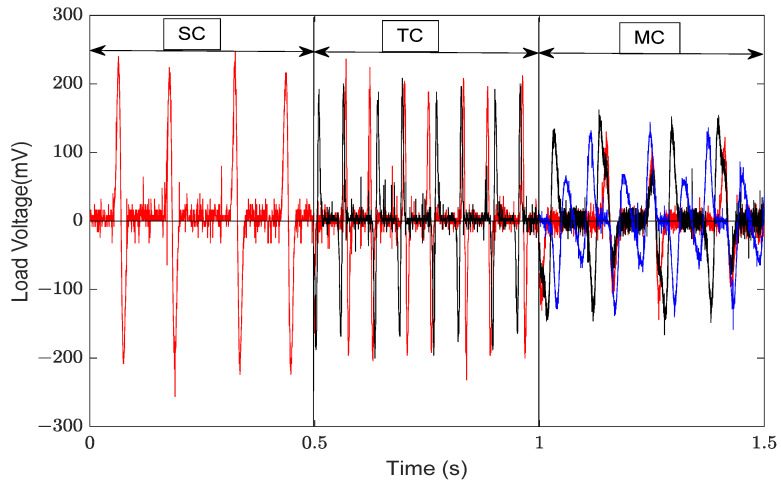
Comparison of output waveforms of SC, TC, and MC at a velocity of 9 m/s.

**Figure 18:**
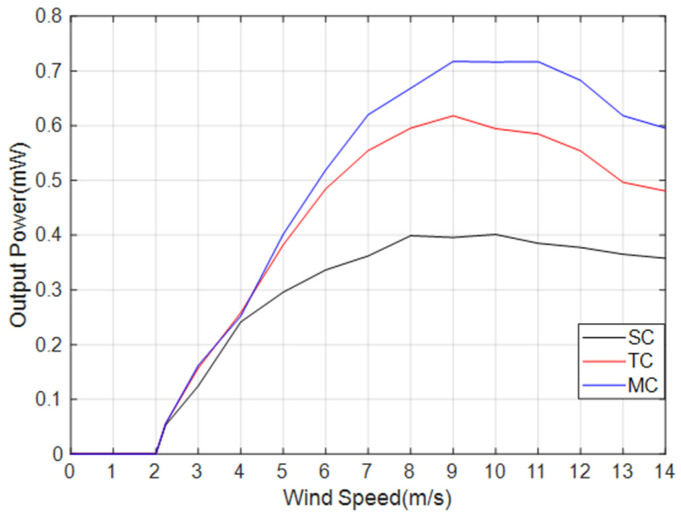
Comparison of output power of the SC, TC, and MC.

According to the aforementioned analysis results, the optimal output of SC, TC, and MC has been chosen for comparison. Figure 17 illustrates the comparison of output waveforms under three distinct conditions, all measured at a wind speed of 9 m/s. Figure 18 depicts the comparison of output power. The greater the number of induction coils, the greater the duty cycle of the MEGEH and the greater the output power. At a wind speed of 9 m/s, the comprehensive output duty ratios for the three cases are 30%, 51%, and 100%, respectively. Additionally, the peak voltage gradually decreases, leading to a smoother output curve. The comprehensive output power values are 0.4 mW, 0.62 mW, and 0.72 mW, respectively. The output power increases by 55% and 16%, respectively. The critical wind speed of the galloping remains consistent due to the constant initial distance between the magnet and the coil.

Therefore, the utilization of the multiple off-center coil arrangement not only reduces the critical wind speed, but also enhances the output power of the energy harvester and improves its output characteristics.

**Figure 19:**
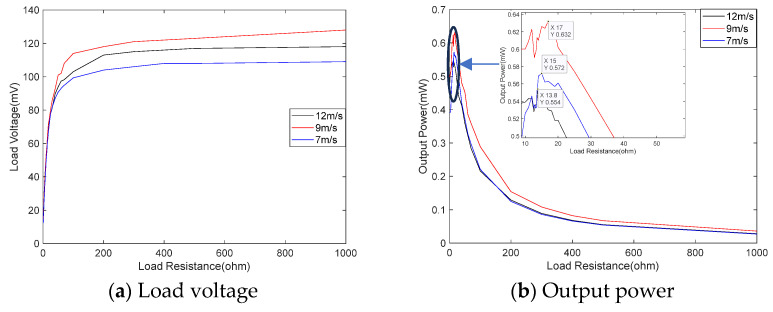
Relationship between output voltage and power with load resistance.

Figure 19 shows the relationship between the output voltage (a) and power (b) and load impedance when the TC is connected in parallel. With the increase in the load impedance, the output voltage continuously increases. When the impedance is less than 20 Ω, the output voltage increases rapidly; when the load reaches 100 Ω, the output voltage increases very slowly. The output power shows a trend of initially increasing and then decreasing. When the load is close to the internal resistance of the coil, the output power is larger. At wind speeds of 12 m/s, 9 m/s, and 7 m/s, the maximum power is 0.554 mW, 0.632 mW, and 0.572 mW, respectively. The corresponding load impedances are 13.8 Ω, 17 Ω, and 15 Ω, respectively. The optimal load is not fixed, and the higher the power, the greater the optimal load. Compared to the output power of 0.55 mW, 0.62 mW, and 0.55 mW under a load of 13.2 Ω, the difference is small. Therefore, the optimal load can be selected by considering the coil’s impedance.

## 4. Conclusions

In this paper, we propose an electromagnetic galloping energy harvester with multiple off-center coils. The arrangement of the coils, both in terms of quantity and position, affects the critical wind speed and output power of the energy harvester. By adjusting the position and quantity of the induction coils, the distribution of the electromagnetic damping force can be modified. This leads to an improved duty cycle and output power of the MEGEH, as well as a reduction in the critical wind speed. Consequently, the overall performance of the MEGEH is enhanced. Adding coils can increase the output of the energy harvester, but the rate of the increase in the output decreases. At a wind speed of 9 m/s, the duty ratios for the SC, TC, and MC are 30%, 51%, and 100%, respectively. The total output powers are 0.4 mW, 0.62 mW, and 0.72 mW, respectively. However, the rate of the output growth decreases from 55% to 16%. The position of the coils affects the initial electromagnetic damping of the energy harvester. Changing the position can reduce the initial electromagnetic damping, thereby decreasing the critical wind speed. The critical wind speed of the MEGEH decreases as the induction coil is positioned further away from the vibration center. When the distance is sufficiently large, the electromagnetic damping force becomes negligible. When the induction coil is placed at the central position, the MEGEH demonstrates its maximum critical wind speed, which is measured at 4.01 m/s. When the initial distance between the induction coil and the vibrating component is increased to 10 mm, the critical wind speed reaches its minimum value of 2.23 m/s. Therefore, it is necessary to optimize the arrangement of the coils. The coils of the MEGEH should be arranged with the MC and a 10 mm offset from the center.

## Figures and Tables

**Table 1:** The MEGEH’s parameters.

Structure	Parameters	Value
Bluff body	h × d × d (mm)	35 × 15 × 15
Magnet	Remanent flux density (T)	0.34
	Diameter(mm)	10
	Thickness (mm)	4
Elastic beam	Young modulus (GPa)	110
	Dimensions (mm)	63 × 10 × 0.15
	Poisson ratio	0.34
	stiffness coefficient K	6.9
Coil	Wire diameter (mm)s	0.2
	Height (mm)	10
	Inner diameter (mm)	10
	Coil turn	300
	Outer diameter (mm)	12.3
	Resistance (Ω)	13.2
Aerodynamic coefficient	A_1_	2.69
	A_3_	−168.4
The gap between the coil and the bluff body	(mm)	1.0

**Table 2:** The amplitude of the energy harvester in the Z-direction when S = 0.

Wind speeds (m/s)	4.08	5	6	7	8	9	10	11	12	13	14
Amplitude (mm)	14.7	18.4	22.7	25.8	27.1	27.7	28.2	28.7	29.6	30	30.3

**Table 3:** Installation position of two sets of coils.

Group	Position
The first group	S_1_ = 3 mm, S_2_ = 11 mm
The second group	S_1_ = 5 mm, S_2_ = 9 mm
The third group	S_1_ = 7 mm, S_2_ = 7 mm
The fourth group	S_1_ = 10 mm, S_2_ = 10 mm

**Table 4:** Comparison of output power between the fourth group and SC when TC is connected in parallel at different wind speeds (mW).

Wind Speed (m/s)	2.23	3	4	5	6	7	8	9	10	11	12	13	14
TC	0.06	0.16	0.26	0.38	0.48	0.55	0.6	0.62	0.59	0.58	0.55	0.5	0.48
SC	0.05	0.12	0.24	0.3	0.34	0.36	0.4	0.4	0.4	0.39	0.38	0.37	0.36

**Table 5:** Installation position of MC.

Group	Position
The first group	S_1_ = 3 mm, S_2_ = 11 mm, S_3_ = 14 mm
The second group	S_1_ = 5 mm, S_2_ = 9 mm, S_3_ = 14 mm
The third group	S_1_ = 7 mm, S_2_ = 7 mm, S_3_ = 14 mm
The fourth group	S_1_ = 10 mm, S_2_ = 10 mm, S_3_ = 14 mm

## Data Availability

Data are contained within the article.
